# Autotaxin expression and its connection with the TNF-alpha-NF-κB axis in human hepatocellular carcinoma

**DOI:** 10.1186/1476-4598-9-71

**Published:** 2010-03-31

**Authors:** Jian-Min Wu, Yan Xu, Nicholas J Skill, Hongmiao Sheng, Zhenwen Zhao, Menggang Yu, Romil Saxena, Mary A Maluccio

**Affiliations:** 1 Department of Surgery, Indiana University School of Medicine, Indianapolis, IN 46202, USA; 2 Department of Obstetrics and Gynecology, Indiana University School of Medicine, Indianapolis, IN 46202, USA; 3 Department of Medicine, Division of Biostatistics, Indiana University School of Medicine, Indianapolis, IN 46202, USA; 4 Clarian Pathology Laboratory, Indiana University School of Medicine, Indianapolis, IN 46202, USA

## Abstract

**Background:**

Autotaxin (ATX) is an extracellular lysophospholipase D that generates lysophosphatidic acid (LPA) from lysophosphatidylcholine (LPC). Both ATX and LPA have been shown to be involved in many cancers. However, the functional role of ATX and the regulation of ATX expression in human hepatocellular carcinoma (HCC) remain elusive.

**Results:**

In this study, ATX expression was evaluated in tissues from 38 human HCC and 10 normal control subjects. ATX was detected mainly in tumor cells within tissue sections and its over-expression in HCC was specifically correlated with inflammation and liver cirrhosis. In addition, ATX expression was examined in normal human hepatocytes and liver cancer cell lines. Hepatoma Hep3B and Huh7 cells displayed stronger ATX expression than hepatoblastoma HepG2 cells and normal hepatocytes did. Proinflammtory cytokine tumor necrosis factor alpha (TNF-α) promoted ATX expression and secretion selectively in Hep3B and Huh7 cells, which led to a corresponding increase in lysophospholipase-D activity. Moreover, we explored the mechanism governing the expression of ATX in hepatoma cells and established a critical role of nuclear factor-kappa B (NF-κB) in basal and TNF-α induced ATX expression. Further study showed that secreted enzymatically active ATX stimulated Hep3B cell invasion.

**Conclusions:**

This report highlights for the first time the clinical and biological evidence for the involvement of ATX in human HCC. Our observation that links the TNF-α/NF-κB axis and the ATX-LPA signaling pathway suggests that ATX is likely playing an important role in inflammation related liver tumorigenesis.

## Background

Hepatocellular carcinoma/cancer (HCC) is one of the most common malignant tumors worldwide [1]. It most often develops in the background of underlying liver disease, such as hepatitis. Historically a challenge for Asian countries, the increasing incidence of hepatitis C has made HCC a major health problem within the United States in recent years [2]. Early stage HCC is potentially curable with liver transplant or resection. However, most patients present with more advanced disease and for these patients' treatment options are limited. Inroads into effective therapies have been thwarted by a gap in our understanding of the molecular mechanisms involved in cancer development and progression within its complex microenvironment. Therefore, studies elucidating the mechanism and signaling pathways involved in HCC development and progression are imperative.

Previous microarray analysis from our laboratory identified autotaxin (ATX) as one a gene with enhanced mRNA expression in human hepatitis associated HCC [3]. Reports from other labs showed that serum ATX activity and plasma lysophosphatidic acid (LPA) level are increased in various liver injuries in rats in relation to their severity [4]. ATX was initially characterized as an autocrine motility factor from A2058 melanoma cell conditioned medium [5]. It has been subsequently shown that ATX acts as an important mediator of tumorigenesis by stimulating angiogenesis, as well as survival, growth, migration, and invasion of tumor cells [6-8]. In particular, recent studies using ATX knockout mice suggest that ATX contributes to tumor progression by stabilizing blood vessels in the vicinity of tumors [9,10]. Although ATX has been showed to affect adhesion through integrin-dependent focal adhesion assembly [11,12], the main impact of ATX on cancer biology is mostly due to its intrinsic lysophospholipase D (lyso-PLD) activity. Through the conversion of lysophosphatidylcholine (LPC) into LPA and to a less degree, sphingosylphosphorylcholine (SPC) into sphingosine-1-phosphate (S1P) [13,14], ATX regulates cell activation by changing signaling induced by LPC versus LPA.

LPA is an important lipid mediator that elicits a broad spectrum of biological effects by activating G protein-coupled receptors (GPCRs). The biological functions of LPA included, but not limited to cell proliferation, migration, platelet aggregation, smooth muscle contraction, and cytoskeletal reorganization. In the context of cancer, LPA could induce stress fiber formation, membrane ruffling, and lamellipodia formation [15-17]. The aberrant ATX expression may lead to altered LPC/LPA balance and their receptor-mediated functions, resulting in enhanced tumor progression. Hence, the molecular events that lead to the aberrant ATX expression and the subsequent abnormal LPA production are significant for understanding the mechanisms involved in cancer progression. In this study, we examined the expression of ATX antigen in HCC tissue using immunohistochemistry. The regulatory mechanism of ATX by the key inflammatory component TNF-α/NF-κB axis was studied in human hepatoma cell lines. We also demonstrated that ATX is involved in the invasive potential of HCC cells.

## Results

### ATX antigen expression in human HCC

**Figure 1:**
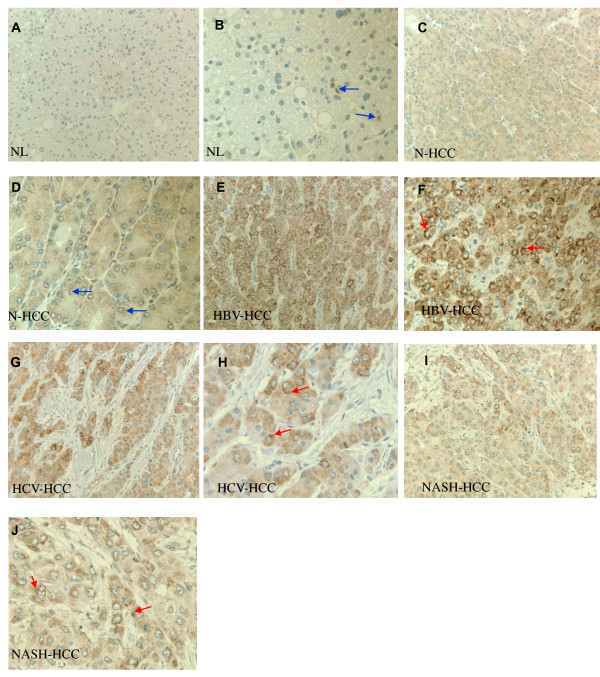
**Representative microphotographs of immunohistochemical analysis of ATX antigen expression in human normal liver and HCC tissue**. **A, B**, normal control liver (NL). **C, D**, HCCs developed from normal liver background with neither inflammatory lesion nor well-established risk factors, and were classified as normal-HCC (N-HCC). **E, F**, Hepatitis B-related HCC (HBV-HCC). **G, H**, hepatitis C-related HCC (HCV-HCC). **I, J**, nonalcoholic steatohepatitis (NASH) related-HCC (NASH-HCC). No detectable ATX antigen expression was observed in hepatocytes of normal liver although a small portion of stromal cells showed weak ATX immunoreactivity (A and B, blue arrow). Intense and widespread ATX immunoreactivity was observed in the cytoplasm of tumor cells in HBV-, HCV- or NASH-associated HCC (E-J, red arrow). Weak ATX immunoreactivity was observed in the tumor cells of N-HCC (C and D, blue arrow). A, C, E, G, and I, original magnification, × 200; B, D, F, H and J, original magnification, × 400. Images were captured with a Leica DM5000 B system (Leica Microsystems).

We have previously examined ATX mRNA expression in human HCC tissues [3]. Here we investigated the ATX protein expression by immunohistochemical approach in tissues form 38 HCC cases and 10 normal controls. The overall positive staining of ATX in normal samples was 20%, with a small portion of stromal cells showed weak ATX immunoreactivity, but not normal hepatocytes (Figure 1A and 1B). In contrast, the overall positive rate of ATX protein expression in HCC was 89% (34 of 38). ATX expression was varied from weak to strong, and the majority of immunoreactivity was heterogeneously distributed in the cytoplasm of tumor cells (Figure 1C-J). Thus, ATX protein expression was significantly increased in HCC tissues when compared with normal liver specimens.

### Correlation between ATX overexpression and clinicopathological parameters in human HCC

Table 1 showed the correlation of ATX levels with clinicopathologic features. Among the 38 HCC samples, 21 (55%) cases were scored 3+, 5 (13%) cases 2+, 8 (21%) cases 1+, and 4 (10.5%) cases 0. Fisher exact test was applied to assess the correlations between ATX expression and clinic pathologic variables of HCC. High expression of ATX was more frequent in HCC with risk factors such as hepatitis compared to the HCCs which were developed from normal liver background with neither inflammatory lesions nor identified risk factors (Normal-HCC) (*P *= 0.0053). A statistical difference in expression of the ATX protein between HCC associated with an inflammatory background and those without inflammatory changes in the adjacent liver was also evident (*P *= 0.0003). In addition, HCC without cirrhosis displayed lower level of ATX expression than those with cirrhosis (*P *= 0.00031). These data have not only confirmed our previous observation at the protein level, but also revealed its association with inflammation, which support the potential role of ATX in the pathogenesis of human HCC.

**Table 1:** Correlations between ATX expression and clinicopathologic variables of 38 patients with HCC.

Clinicopathologic variables		ATX expression				P
			
	Subtotal	0	1+	2+	3+	
**Age**						P = 1
< = 50	6	1	1	1	3	
>50	29	3	4	6	16	

Gender						P = 0.836
Male	32	3	7	5	17	
Female	6	1	1	0	4	

**Etiology**						**P = 0.0053**
HBV	7	0	0	0	7	
HCV	11	0	2	1	8	
NASH	10	0	3	2	5	
Normal-HCC	10	4	3	2	1	

**Inflammation**						**P = 0.00031**
Without	10	4	3	2	1	
With	28	0	5	3	20	

**Tumor grade**						P = 0.932
I	22	2	4	3	13	
II	9	2	3	1	6	
III	4	0	1	1	2	

**Lymphovascular invasion**						P = 0.414
Absence	26	2	5	4	15	
Presence	9	2	3	1	3	

**Tumor nodule no.**						P = 0.927
Multiple(> = 2)	8	1	1	1	5	
Solitary	27	3	7	4	13	

**Serum AFP (ug/L)**						P = 0.3585
< = 20	22	1	6	3	12	
>20	7	1	0	1	5	

**Liver cirrhosis**						**P = 0.0003**
Absence	10	4	3	2	1	
Presence	28	0	5	3	20	

**Note**: The expression of ATX was determined in 38 cases of HCC by immunohistochemical studies as described in Materials and Methods. The correlations between ATX expression and clinicopathologic variables of HCCs were evaluated by the Fisher exact test. Statistically significant values are in bold. Some clinicopathologic factors only included 35 or less cases information as several cases missed part of the clinicopathologic information.

### Differential expression of ATX in human liver cell lines

**Figure 2:**
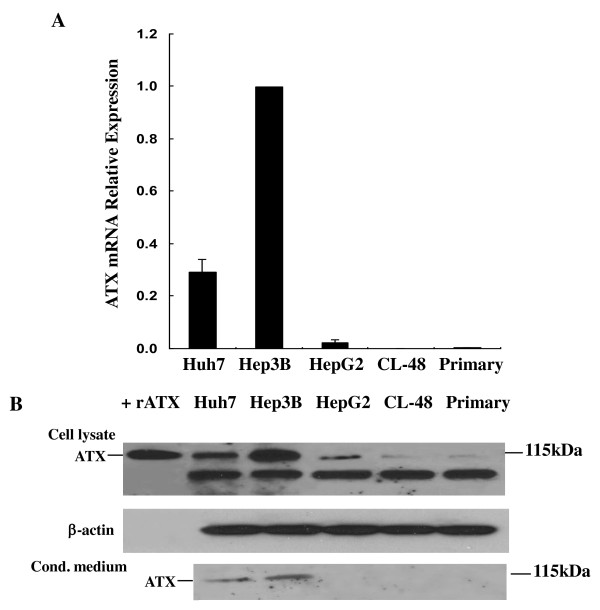
**Differential expression of ATX in human liver cancer cell lines (Huh7, Hep3B and HepG2), normal embryonic liver cell line CL-48, and normal primary hepatocytes**. **A**. Comparison of ATX mRNA levels by qRT-PCR analysis. Columns are average ± SD from three independent experiments. **B**. Equal number of cells (4 × 10^5^) were plated into 100 mm dishes and incubated in serum free EMEM containing 0.1% BSA for 24 hours. Cells were lysed with RIPA buffer and 15 μg of lysates was used for SDS-PAGE and probed for ATX antibody, β-actin level was used as a loading control (top two pannels). 1/10 volume of concentrated medium was used for immunoblot (bottom panel). An intense band of 110 kDa was detected in the cell lysate from Hep3B and Huh7 cells, which is corresponding to the positive ATX control. The sizes of the MW markers are shown on the right. rATX indicates recombinant ATX protein which is a positive control. IB indicates immunoblot.

To gain a better insight into the expression and function of ATX in HCC, we examined the expression of ATX in three human liver cancer cell lines (Hep3B, Huh7 and HepG2), a human normal embryonic liver cell line CL-48, and human normal primary hepatocytes. As shown in Figure 2A, quantitative real time RT-PCR (qRT-PCR) revealed that ATX expression in Hep3B and Huh7 cells was 42- and 14-folds higher than that of HepG2 cells, respectively, while the expression of ATX mRNA in CL-48 was negligible when compared with that of Hep3B cells (AvgCt ~37.5 vs. AvgCt ~21.0). Human normal primary hepatocytes also displayed very low level of ATX mRNA (AvgCt ~32.4). ATX mRNA expression levels in these cells were well-correlated to its protein expression levels determined by immunoblot analysis. ATX protein expressed at higher levels in Hep3B and Huh7 cells than in HepG2 cells. The ATX protein levels in CL-48 cells and normal primary hepatocytes were even lower than that in HepG2 cells (Figure 2B, the top panel). Since ATX protein can be secreted from cells, and the soluble ATX has been considered to be mainly responsible for extracellular LPA production (12), we measured secreted ATX levels in cultural media from these cells. Only Hep3B and Huh7 produced measurable amounts of secreted ATX in their conditioned media (Figure 2B, the bottom panel). These results are consistent with our immunohistologic studies shown in Figure 1, suggesting aberrant ATX expression and regulation in human HCC.

### ATX expression and secretion were selectively promoted by proinflammatory cytokine TNF-α

**Figure 3:**
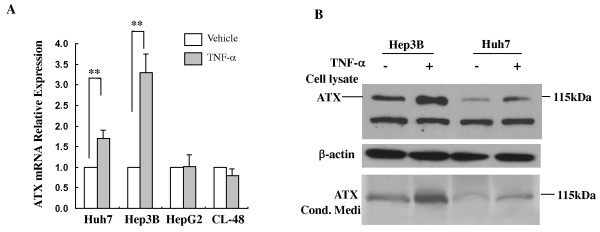
**TNF-α induces ATX expression and secretion selectively in hepatoma cell lines Hep3B and Huh7**. **A**. Effect of TNF-α on the expression of ATX. Serum starved cells were treated with or without TNF-α (10 ng/ml) for 16 hours. Results are the mean ± SD from three QRT-PCR experiments. **, *P *< 0.01. **B**. Starved Hep3B or Huh7 cells were treated with TNF-α (10 ng/ml) for 20 hours. Cell culture medium and cell lysates were collected. 15 μg cell lysate were used for immunoblot analysis. IB indicates immunoblot.

The expression of ATX is regulated by growth factors and cytokines. For example, FGF and EGF have been shown to induce ATX expression, whereas certain cytokines such as interleukin-1 (IL-1), IL-4 and interferon-gamma (IFN-γ) decrease the expression of ATX mRNA in cultured fibroblast-like synoviocytes (SFC) [18]. Inflammatory cytokines are known to be associated with the inflammation related liver diseases [19]. Here we examined the effect of a prototype inflammatory cytokine, TNF-α on the expression of ATX in human liver cell lines. Assessed by QRT-PCR assays, TNF-α engagement increased ATX mRNA levels more than 3-fold and 1.7-fold in Hep3B cells and Huh7 cells, respectively. In contrast, TNF-α did not affect ATX expression in either HepG2 or CL-48 cells (Figure 3A). The stimulatory effect of TNF-α on cellular and secreted ATX protein expression was further demonstrated by immunoblot analyses (Figure 3B).

### Up-regulation of ATX induced by TNF-α was associated with increased Lyso-PLD activity by conversion of LPC into LPA

**Figure 4:**
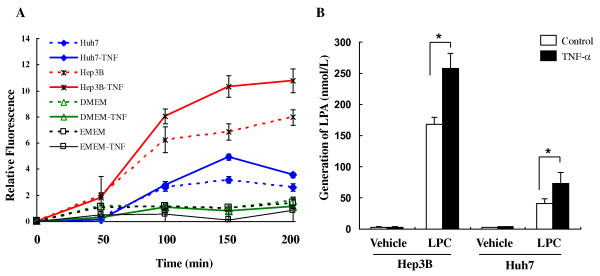
**Up-regulation of ATX induced by TNF-α is associated with increased lysophospholipase D (lyso-PLD) activity by conversion of LPC into LPA in Hep3B and Huh7 cells**. Serum starved Hep3B or Huh7 cells were treated with TNF-α (10 ng/ml) or vehicle (0.1%BSA/PBS) for 20 hours. **A**. Conditioned media (CM) or control media (DMEM or EMEM) were concentrated (40-fold) and assayed for ATX activity using the FS-3 compound. The results are shown as the average of relative fluorescence activity ± SD from three experiments. **B**. CM were incubated with 15 μM LPC (18:1) for 3 hours at 37°C. Lipids were analyzed by liquid chromatography-mass spectrometry (LC-MS). Results are level of LPA (18:1) from three experiments and presented as mean ± SD. *, *P *< 0.05.

Being an enzyme with lyso-PLD activity, ATX plays a critical role in LPA production [13]. In order to explore whether TNF-α-induced ATX led to a corresponding increase in ATX/lyso-PLD activity, we collected conditioned media from Hep3B and Huh7 cells that were treated with TNF-α or vehicle (0.1%BSA/PBS). ATX/lyso-PLD activity in conditioned media was measured with fluorescent LPC analogue FS-3 as substrate [20]. The basal level of lyso-PLD activity secreted by Hep3B cells was higher than that from Huh7 cells. After TNF-α stimulation, both Hep3B and Huh7 exhibited a ~1.5-fold increase of secreted lyso-PLD activity (*P *< 0.01, Figure 4A), indicating that TNF-α was able to increase lyso-PLD activity in cell culture media by inducing ATX expression. We next checked the LPA production by incubating the conditioned media with 15 μM LPC (18:1), a lyso-PLD substrate, followed by liquid chromatography/Mass spectrometry (LC-MS) analysis. The media from either vehicle- or TNF-α-treated Hep3B cells contained low levels of LPA (18:1) (2.52 ± 1.39 and 2.93 ± 0.53 nmol/L, respectively). After supply of ATX substrate (LPC), relatively high levels of LPA (167.6 ± 11.8 nmol/L 18:1-LPA) were detected in the conditioned medium of Hep3B cells. TNF-α treatment further elevated LPA levels by more than 1.5-fold (257.7 ± 24.79 nmol/L 18:1-LPA, *P *< 0.05). In parallel, more LPC was hydrolyzed in TNF-α treated group than control group (data not shown). A similar effect was observed in Huh7 cells, where TNF-α treatment induced an approximately 1.8-fold increase of LPA generation (73.15 ± 18.1 nmol/L 18:1-LPA, *P *< 0.05), albeit the absolute LPA levels were lower (Figure 4B). Thus, our results demonstrated that secreted ATX from Hep3B or Huh7 cells had lyso-PLD activity and TNF-α induced ATX expression, secretion, and lyso-PLD activity, resulting increased extracellular LPA production from LPC. Because Hep3B cells express higher level of both ATX mRNA and ATX protein when compared with Huh 7 and HepG2, we mainly focused on Hep3B cell line for more in-depth studies hereafter.

### Secreted enzymatically active ATX promoted Hep3B cellinvasion

**Figure 5:**
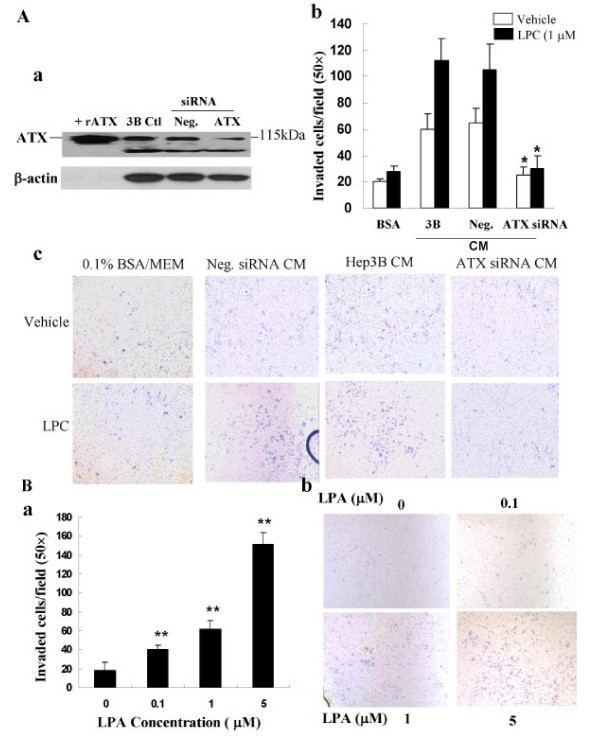
**Secreted enzymatically active ATX promoted Hep3B cell invasion**. **A**. ***a***, Hep3B cells were transfected with ATX siRNA or negative siRNA, and ATX expression was examined by immunoblot. ***b***, serum starved Hep3B cells were chemoattracted by MEM/0.1% fatty acid-free BSA or conditioned media (CM) plus or minus 1 μM LPC (18:1). Neg. CM, CM from Hep3B cells transfected with negative siRNA. 3B CM, CM from Hep3B cells; ATX siRNA CM, CM from Hep3B cells transfected with ATX siRNA. Each sample was tested in triplicate and results are reported as mean ± SD of invaded cells of two independent experiments, bars, SD. *, *P *< 0.05. ***c***, representative fields of invaded and stained cells. **B. *a***, starved Hep3B cells were chemoattracted by various doses of LPA and representative fields of invaded cells are shown in ***b***. **, *P *< 0.01.

ATX has been shown to stimulate the migration and invasion of a variety of cells types, including cancer cells, fibroblasts, and vascular smooth muscle cells [7,21]. We next determined whether ATX secreted by Hep3B cells acted as a source of chemoattractant by utilizing invasion/migration assays. As shown in Figure 5A, Hep3B conditioned medium induced ~3-fold higher rate of Hep3B cells invasion than EMEM/BSA did (*P *< 0.05), suggesting an autocrine action. Specifically knockdown of ATX in Hep3B cells resulted in significantly reduced chemotactic potential of the conditioned medium from Hep3B cells. To further elucidate whether ATX-LPA linked to the invasion of Hep3B cells, we evaluated Hep3B cell invasion in the presence and absence of exogenous LPC (18:1). We found that addition of LPC (1 μM) to EMEM/BSA did not induce invasion of Hep3B cells. However, addition of LPC to Hep3B conditioned medium resulted in a 1.8-fold greater rate of invasion above the vehicle control (Figure 5A, b, *P *< 0.05). In addition, it was noted that the presence of LPC in conditioned medium from ATX siRNA-transfected Hep3B cells failed to induce Hep3B cells invasion. These data suggest that LPC itself is not a chemoattractant, and an ATX-dependent action is required for the activity. To directly test whether LPA is the molecule that involved in the invasion process, we tested the effect of LPA on cell invasion in Hep3B cells. LPA was found to dose-dependently induced the invasion of Hep3B cells, which peaked at 5 μmol/L (Figure 5B). These data suggest that ATX-mediated conversion of LPC to LPA is critical for cell invasion.

### Nuclear factor-kappa B (NF-κB) mediated the basal and TNF-α induced ATX expression

**Figure 6:**
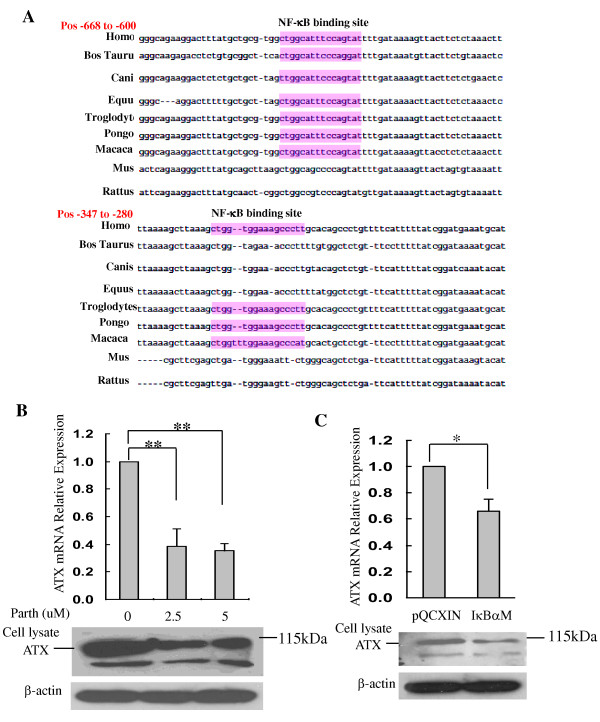
**NF-κB mediates the basal expression of ATX**. **A**. Alignment of the ATX promoter region 2000 nt upstream of the transcription start site from 9 eutherian mammals. Two highly conservative consensus sequences for NF-κB binding sites are identified. **B**. Starved Hep3B cells were treated with parthenolide (0, 2.5 or 5 μM) for 16 hours. **C**. ATX expression in stable cell line Hep3B-IκBαSR which NF-κB activity was blocked by mutant IκBα (IκBαSR). Vector infected cell line Hep3B-pQCXIN served as control; IκBαSR indicates Hep3B-IκBαSR cells; pQCXIN indicates Hep3B-pQCXIN cells. ATX mRNA expression was determined by qRT-PCR (top panel in B, C), Values are the mean ± SD of three experiments. ** *P *< 0.01, * *P *< 0.05. ATX protein expression was determined by immunoblot (IB) (Bottom panel in B, C). β-actin expression was used as a loading control.

**Figure 7:**
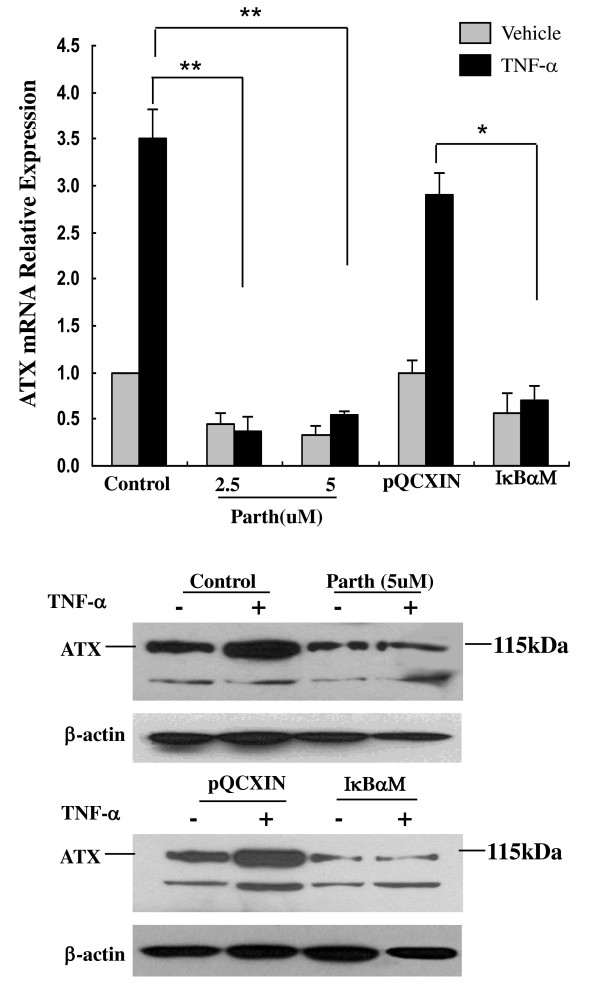
**NF-κB mediates TNF-α-induced expression of ATX**. Parthenolide pretreated Hep3B cells (4 hours), and stable cell lines Hep3B-IκBαSR and Hep3B-pQCXIN were stimulated with or without 10 ng/mL human TNF-α for 16 hours. ATX mRNA expression was determined by qRT-PCR (top panel), Values are the mean ± SD of three experiments. ** *P *< 0.01, * *P *< 0.05. ATX protein expression was determined by immunoblot (IB) (Bottom panel). β-actin expression was used as a loading control.

Activation of TNF-α/NF-κB pathway has been shown to contribute to inflammation-associated cancer such as hepatitis-related HCC [22]. To determine whether NF-κB is involved in the regulation of ATX expression, ATX promoter region analyses were performed using TFSEARCH: Searching Transcription Factor Binding Sites (ver 1.3) http://www.cbrc.jp/research/db/TFSEARCH.html and AliBaba 2.1 http://www.gene-regulation.com/pub/programs/alibaba2/index.html. Further analyses were performed using ConTra promoter alignment analysis tool (http://www.dmbr.ugent.be/prx/bioit2-public/ConTra/index.php. Stringency: core = 0.95, similarity matrix = 0.85). Two highly conservative consensus sequences for NF-κB binding sites were identified in ATX promoter region 2000 nt upstream of the transcription start site from 9 eutherian mammals (Figure 6A) [23,24]. To evaluate the contribution of NF-κB to basal transcription of ATX, the NF-κB activity was first inhibited pharmacologically by a soluble inhibitor parthenolide [25]. QRT-PCR results showed that parthenolide (2.5 μM and 5 μM) treatment inhibited ATX expression by 62% and 65% respectively (Figure 6B top panel). To further confirm the role of NF-κB, we determined the effect of NF-κB activity on expression of ATX using a stable cell line with reduced NF-κB activity. This cell line over-expresses an inhibitor of kappaB alpha (IκBα) mutant (S32A and S36A) (IκBαSR) and has been characterized in our lab recently [26]. A significant reduction of ATX expression compared to the vector control cell line (Hep3B-pQCXIN) was observed in the mutant (S32A and S36A) IκBαSR cell line (Figure 6C). Immunoblot analyses confirmed that both parthenolide and overexpression of IκBαSR reduced ATX expression at the protein level. Since NF-κB is known as a main target transcription factor of TNF signaling [27], we next determined whether NF-κB participates in the induction of ATX by TNF-α. Hep3B cells were stimulated with TNF-α in the presence or absence of parthenolide. Pretreatment with parthenolide prevented the induction of ATX by TNF-α at both mRNA and protein levels (Figure 7). Expression of IκBαSR also blocked TNF-α induced ATX expression in Hep3B-IκBαSR cells. Together, these data indicate that NF-κB activity plays a key role in the regulation of basal and TNF-α induced ATX expression.

## Discussion

This is the first report to study the expression and the functional roles of ATX in human HCC. We showed for the first time that ATX protein was over-expressed in human HCC tissues compared with that in normal controls. Enhanced expression of ATX in HCC is significantly correlated with liver inflammation, cirrhosis, as well as risk factor such as hepatitis. ATX was also over-expressed in human hepatoma cell lines Hep3B and Huh7 cells compared to hepatoblastoma HepG2 and normal hepatocytes.

ATX-null mice embryos failed to develop into mature vessels and died at E11.5 [9]. While ATX over-expression was presented in various cancers and promotes tumor progression by stimulating angiogenesis, tumor cell survival, growth, migration and invasion [6-8]. Recently, Mills's lab demonstrated that expression of ATX or LPA receptor in mammary epithelium of transgenic mice contributes to the initiation and progression of estrogen receptor (ER)-positive, invasive, and metastatic mammary cancer [28]. Our observations showing the overexpression of ATX in HCC tissues and cell lines, as well as its relative low levels in normal liver cell lines and tissues imply its important role in both liver physiological and pathological activities.

Being the key enzyme with lyso-PLD activity, the aberrant expression of ATX has the potential to alter the delicate balance between LPA signaling and LPC signaling in the local liver microenvironment. LPC is phospholipid with both proinflammatory activity and immunoregulatory activity by stimulating the expression of a serial of genes, including NO synthase, monocyte chemoattractant protein-1 (MCP-1), inter-cellular adhesion molecule (ICAM-1), vascular cell adhesion molecule-1 (VCAM-1) and growth factors in endothelial cells [29-33]. In addition, LPC has been reported to promote vascular smooth muscle cell proliferation, attract monocytes, inhibit endothelium dependent relaxation, reduce endothelial cell migration, and promote the development of mature dendritic cell [34-37]. Moreover, LPC is also required for the cytotoxic response of human NK cells to tumor cells [38]. On the other hand, LPA mediates a broad range of biological activities such as wound healing, vascular remodeling, and cell migration and survival. LPA and its analogs were also proposed to be critical endogenous mediators that regulate survival, motility, proliferation, and differentiation of oval cell/hepatocyte progenitors in liver regeneration [39]. Oval cell proliferation was proposed to be associated with an increased risk for development of HCC with advancing liver disease, particularly when cirrhosis is present [40]. Serum ATX activity and plasma LPA level were increased in chronic hepatitis C in association with liver fibrosis [41]. Interestingly, we also found that ATX over-expression in HCC was associated with liver cirrhosis. The aberrant production of LPA may bind to its receptors and results in the altered activation of LPA signaling pathways, including, but no limited to activation of the PI3K-AKT, Ras/MEKK/MAPK, p38 MAPK, and JNK pathways. These signaling pathways have been shown to be actively involved in HCC development by controlling angiogenesis, cell motility, cell proliferation and survival [42,43]. Therefore, the aberrant expression of ATX along with the consequently abnormal production of LPA in the liver microenvironment may fuel the process of liver carcinogenesis.

Chronic inflammation has long been associated with the development of liver cancer. Three lines of evidence obtained from the current study support a link between ATX expression/function to inflammation in liver diseases. First, our immunohistochemistry data from human liver tissue showed the differential expression of ATX in HCC with different etiologies. Hepatitis literally means inflammation of the liver, and is the major cause of HCC. ATX expression in hepatitis-related HCC tissues is significantly elevated compared to those HCC tissues developed from non-cirrhotic "non-inflammatory" background which indeed show no signs inflammatory cell infiltration as we observed in the samples from patients with chronic active hepatitis or steatohepatitis. Secondly, the ATX expression levels correlated well to the derivative origins in HCC cell lines related to inflammation. Hep3B cells were derived from a patient with hepatitis and therefore may have unique response systems that are associated with the inflammatory background of a hepatitis infected liver [44]. Rice lab showed that Huh7 cells had a favorable cellular environment for hepatitis C virus replication [45]. We use it as a second cell line that may respond to inflammatory mediators thereby calling attention to unique signals associated with inflammatory associated cancers. But we need to be cautious on this data since Huh 7 cells are not well characterized although they were derived from a Japanese patient with well differentiated HCC [46]. In contrast, HepG2 is derived from a human hepatoblastoma which almost always arise in an otherwise normal liver and is most unlikely to be associated with inflammation [47,48]. Hep3B and Huh7 cells, but neither HepG2 nor normal hepatocytes exhibit enhanced expression of ATX. As we are focusing on the mechanisms that potentially overlap between different etiologies of inflammatory induced HCC, whether there are any potential associations between viral antigens and the ATX/LPA pathway remain to be further studied. Finally, TNF-α, a pro-inflammatory cytokine, further promoted ATX secretion and LPA production in Hep3B and Huh7 cell lines. Moreover, the secreted enzymatically active ATX promoted Hep3B cell migration/invasion, which is dependent on extracellular LPC concentration and can be directly demonstrated by LPA's effect. Our mechanistic studies show that NF-κB activity is important for TNF-α 's activity. Our previous work showed NF-κB is constitutively activated in Hep3B, HepG2 and CL-48 cell lines, but Hep3B has the strongest basal NF-κB activity among these cell lines [26]. The differential responsiveness of these cell lines to TNF-α in ATX stimulation suggest that these cells may also have differential signaling properties in responding to TNF-α, which remains to be further investigated.

## Conclusions

Taken together, we have shown for the first time the clinical and biological significance of ATX in human HCC. We have also demonstrated for the first time a novel regulation mechanism of ATX expression in human liver cancer cells. The connection between TNF-α/NF-κB pathway and ATX signaling provides new insight into the molecular pathways involved in HCC pathogenesis and indicates that ATX may play a potential role in the connection between inflammation and tumorigenesis in the complex liver microenvironment. Whether this influences initiation, promotion, or metastatic potential remains to be further studied. Since inflammation is the most potent risk factor for human HCC, these findings are highly significant for this research field.

## Methods

### Reagents

TNF-α, parthenolide and fatty acid-free BSA were purchased from Sigma (St. Louis, MO). LPC (1-oleoyl) was obtained from Avanti Polar Lipids, Inc. (Birmingham, AL). ATX activity assay reagents were from Echelon Biosciences, Inc. (Salt lake City, UT, USA). Purified recombinant ATX protein and rabbit polyclonal antibody against ATX and were generous gifts from Dr. Timothy Clair (National Cancer Institute, Bethesda, MD); the polyclonal antibody against ATX was prepared by immunization of rabbits with the peptide ARVRDIEHLTSLDFFRK.

### Human liver tissue and cell lines

This study was approved by Indiana University Institutional Review Board. Liver tumor tissue was collected from patients undergoing resections for HCC at Indiana University Hospital. Normal tissue (n = 10) was obtained from patients undergoing non-liver disease related surgeries. The fresh tissue was formalin fixed, and paraffin embedded for immunohistochemistry. Thirty-eight HCC cases were applied for this study. Eleven of them had HCV infection, seven had HBV infection and ten had non-alcohol steatohepatitis (NASH), as confirmed by serological testing or PCR testing of benign or tumor DNA. Another ten cases of HCC samples were observed in non-cirrhotic liver and they developed in an otherwise normal liver and without identified risk factors. No pathological evidence of inflammatory infiltrates within the background liver was identified, and here they are named as normal-HCC.

CL-48, HepG2 and Hep3B cell lines were obtained from American Tissue Culture Collection and were cultured in Eagle's Minimum Essential Medium (EMEM) with 10% fetal bovine serum at 37°C, 5% CO2. Huh7 cell line was a generous gift provided by Dr. Charles M. Rice's lab (Rockefeller University, New York, NY) and was cultured in Dulbecco's Modified Eagle's Medium (DMEM) with 10% fetal bovine serum at 37°C, 5% CO2. Human normal primary hepatocytes were purchased from Lonza (Lonza, Walkersville, MD) and maintained in hepatocyte culture medium (Lonza, Walkersville, MD). Cells were serum-starved overnight and then treated with TNF-α (10 ng/ml) or parthenolide in serum free media containing 0.1% BSA. Total RNA was extracted or cell lysate was prepared after stimulation for the indicated time.

### siRNA transfection

Small interfering RNA (siRNA) duplexe targeting human ATX and negative control siRNA were purchased from Ambion (Austin, TX). Cells were cultured to 60-70% confluency and then transfected with 10 nM of ATX siRNA or 30 nM negative siRNA using Transfection siPORT™ NeoFX™ kit (Ambion) according to the manufacturer's recommendations. Transfected cells were incubated at 37°C and 5% CO2 for 68-72 hours. Cells were harvested for total RNA or protein preparation and conditioned media were collected for invasion assay.

### Conditioned media and cell extracts preparation andimmunoblot analysis

Conditioned media were prepared by incubating 70% confluent cells in 100 mm dishes for 24 hours in serum-free MEM or DMEM containing 0.1% fatty acid-free BSA. Conditioned media were harvested, clarified by centrifugation, and filtered through a 0.22 μm filter. The conditioned media were concentrated by Amicon Ultra-15 Centrifugal Filter Units before using for immunoblot. At the same time, total cell extracts were prepared from cell monolayer incubated in RIPA buffer (50 mM Tris-HCl, pH 7.4; 150 mM NaCl; 2 mM EDTA, 1 mM sodium orthovanadate, 1% NonidetP40; 1% sodium deoxycholate; 0.1% sodium dodecylsulfate (SDS), 2 mM phenylmethylsulfonylfluoride (PMSF) and protease inhibitor cocktail. Fifteen micrograms of total cellular protein was resolved by SDS-PAGE. Blots were probed with appropriate antibodies. Anti-β-actin was used for loading control.

### Quantitative real time RT-PCR (qRT-PCR)

Total RNA was isolated from cells using the RNeasy kit following the manufacturer's instructions (Qiagen, Valencia, CA). 2 μg total RNA was reverse-transcribed in a total reaction volume of 20 μl using the high capacity cDNA reverse transcriptase kit (Applied Biosystems, Foster City, CA) as described by the manufacturer. Single stranded cDNA products were then analyzed by real-time PCR using standard commercially available TaqMAN probes for ATX (Hs00196470_m1). The amount of target gene was normalized to the internal standard 18S rRNA (Hs99999901_s1) levels and reported as a relative value.

### ATX/lyso-PLD activity assay

The conditioned serum-free medium from Hep3B and Huh7 cells with or without TNF-α stimulation was concentrated (40-fold) using Amicon Ultra 50,000 (Millipore). EMEM and DMEM without cells were used as control. ATX/lyso-PLD activity in concentrated conditioned media was analyzed using fluorogenic substrate FS-3 according to the manufacture protocol. Briefly, 10 ul concentrated medium was mixed with 5 uM FS-3 and assayed in 96-well plate. The change of fluorescent intensity was measured by SpectraMax Gemini EM Fluorescence Microplate Reader (Molecular Devices, Sunnyvale, CA, USA) with excitation and emission wavelengths of 485 and 528 nm, respectively.

### Lipid extraction and analyses

Lipids were extracted from conditioned media and analyzed using LC-MS (API-4000, Applied Biosystems) [49,50]. Briefly, conditioned media were incubated with 15 μM LPC (18:1) for 3 hrs at 37°C. 1.3 mL samples were mixed with 3 mL of MeOH/chloroform (2:1) following the addition of 10 μL of 14:0 LPA (1 μM) as an internal standard and 10 μL of HCl (6N). The samples were vortexed for 1 min and incubated on ice for 10 min. Chloroform (1 mL) and PBS (1 ×) (0.5 mL) was added to separate the phases and samples were vortexed for 1 min prior to centrifugation (1,750 g for 10 minutes, at 10°C). The lower phase was transferred to a new glass tube. The upper phase was re-extracted using 2 mL chloroform and combined with the lower phase. After evaporating the solvent under nitrogen at room temperature, the dried lipids were re-suspended respectively in 100 μl of MeOH and 10 μL of sample will be used for Mass spectrometry (MS) analyses. Typical operating parameters for MS will be as follows: nebulizing gas (NEB) 15, curtain gas (CUR) 8, collision-activated dissociation (CAD) gas 35, electrospray voltage 5000 with positive-ion MRM mode, and a temperature of heater at 500°C. Precursor mode 153 will be set as the daughter ions of LPA. In MRM mode, negative monitoring ions will be at m/z 435 (the parent ion)-153 (the product ion) for 18:1 LPA. The dwell time in the MRM mode will be 75 ms. A TARGA C18 5 μM, 2.1 mm ID× 10 mm TR-0121-C185 (Higgins Analytical, Southborough, MA USA) HPLC column was used for the separation of different phospholipids and for the detection of LPAs. The mobile phase A was MeOH/water/NH4OH (90:10:0.1, v/v/v). The HPLC separations will be 12 min/sample using the following scheme: 1) 100% A for 3 min with a flow rate at 0.2 mL/min; 2) the mobile phase will be changed from 100% A to 100% B in 2 min with a flow rate from 0.2 to 0.8 mL/min; 3) a constant flow rate of 0.8 mL/min for 5 min; 4) the mobile phase will be changed from 100% B to 100% A in 1 min with a flow rate from 0.8 to 0.2 mL/min; and 5) constant flow rate of 0.2 mL/min for 1 min.

### Cellular migration/invasion assays

According to previously described methods [26], invasion assay was performed by using BD BioCoat™ Matrigel™ 24-well invasion chamber (8 μM pore size). In brief, cells were serum-starved overnight and re-suspended into serum free MEM containing 0.1% fatty acid-free BSA. 5 × 10^4 ^cells were added to the top insert, and 750 μl of conditioned medium with or without 1 μM LPC (18:1) was added to the bottom chamber. To determine the effect of LPA on the invasion, serum-free MEM containing 0.1% fatty acid-free BSA with or without LPA (0, 0.1, 1, 5 μM) were added to the bottom chamber. After 24 hours incubation at 37°C in a CO2 incubator, non-invaded cells were removed from the upper surface of the filter with the cotton swab; cells that migrated through the gel insert to the lower surface of the membrane were fixed with 100% methanol, stained with 1% Toluidine blue and counted using a light microscope at 50 × magnification. Each sample was tested in triplicate at least in two independent assays. Results were expressed as mean cell number per field ± SD.

### Immunohistochemistry

Serial 5-micron thick sections of formalin-fixed paraffin embedded tissue were cleared with xylene and rehydrated through graded ethanol and finally immersion in distilled water. Slides were then rinsed in Tris-buffered saline (TBS). Antigen retrieval was performed by using the Dako Target Retrieval kit (Dako, Carpinteria, CA) containing a citrate buffer (pH 6.0) for 10 min at 95°C. Dako's Avidin Biotin blocking system was used for 10 min, and the tissue sections were then rinsed with TBS. The nonspecific binding sites were blocked by incubating with Dako's Protein Block for 10 min. Tissue sections were then incubated with the polyclonal rabbit antibody against ATX (7.8 μg/ml) overnight at 4°C. After washing with TBS, the secondary antibody, Dako Link (Dako LSAB2 kit) was applied for 20 min and then rinsed with TBS. Additional washing was followed by incubation with streptavidin horseradish peroxidase (Dako Label, LSAB2 kit) for 20 min. Immunoreactivity was visualized by incubation of sections with 3, 3'-diaminobenzidine in the presence of hydrogen peroxide. Sections were counterstained with light hematoxylin and mounted with a coverslip. All of the procedures were performed at room temperature except the primary antibody incubation. Microscopic fields evaluated and scored were those with the highest degree of immunoreactivity ("hot spots"). Five fields (40 × fields) per section were analyzed. An intensity score was assigned to each case on a scale from negative to high (0: no staining; +1: weak staining; +2: moderate staining; and +3: strong staining).

### Statistical analysis

Data are presented as means ± SD. Analysis of the significance of differences between two groups was performed by two tailed student's t-test using Instat software (GraphPad, San Diego, CA). P-values of < 0.05 were considered statistically significant. Fisher's exact test was used for the ATX immunoreactivity analysis, and P value < 0.05 was deemed significant.

## Competing interests

The authors declare that they have no competing interests.

## Authors' contributions

MAM supervised and coordinated the study and revised the manuscript. JMW designed the study, performed all the experiments, analyzed the data and prepared the manuscript. YX revised the manuscript and contributed to the LC-MS assay. ZZ performed the LC-MS assay. JS and HS reviewed the manuscript. RS contributed to human tissue acquisition, specimen pathology reviewing and immunohistochemistry data analysis. MY performed the statistic analysis of immunohistochemistry data. All authors read and approved the final manuscript.
